# Effect and Mechanism of Red Mud on the Aging Resistance of Asphalt

**DOI:** 10.3390/ma19061116

**Published:** 2026-03-13

**Authors:** Jiandong Wu, Yuechao Zhao, Jianxiu Sun, Jizhe Zhang, Run Xu, Hongya Yue

**Affiliations:** 1 Shandong Provincial Communications Planning and Design, Institute Group Co., Ltd., Jinan 250101, China; jdwu_mail@163.com (J.W.); sunjianxiu1991@163.com (J.S.);; 2 School of Materials Science and Engineering, University of Jinan, Jinan 250022, China; 3 School of Qilu Transportation, Shandong University, Jinan 250002, China

**Keywords:** asphalt, red mud, aging resistance, mechanism

## Abstract

The primary objective of this study is to investigate the effect and mechanism of replacing limestone powder with red mud as a filler on asphalt aging resistance. The microstructure and porosity characteristics of limestone powder, Bayer process red mud, and sintered red mud were analyzed. Asphalt mastics were then prepared using these fillers. The effect of red mud on the aging resistance of asphalt was evaluated by comparing the conventional physical properties, rheological behavior, and functional groups of the asphalt mastics before and after aging. Fourier transform infrared spectroscopy (FTIR), gel permeation chromatography (GPC), and ultraviolet-visible spectroscopy (UV-Vis) were further employed to elucidate the underlying anti-aging mechanisms. The results indicate that the asphalt mastic containing 4% sintered red mud exhibits the strongest resistance to both thermo-oxidative and UV aging. It shows the lowest increments in softening point, viscosity aging index, and complex modulus aging index, with performance comparable to a commercial anti-aging agent. FTIR and GPC analyses reveal that sintered red mud selectively adsorbs light asphalt components, thereby inhibiting their conversion into heavier fractions during thermo-oxidative aging. UV-vis analysis demonstrates that sintered red mud provides effective UV shielding within the asphalt mastic, substantially mitigating UV-induced damage. In summary, the incorporation of 4% sintered red mud can significantly delay both thermo-oxidative and UV aging processes in asphalt mastics, thereby effectively enhancing the aging resistance of asphalt pavement.

## 1. Introduction

Road infrastructure plays a crucial role in connecting regions, facilitating circulation, and improving the quality of life [1,2]. With ongoing economic and technological advancement, there is a growing demand for sustainable and durable road construction [3,4]. However, the inherent aging tendency of asphalt, an organic binder, poses a significant challenge to the goal of long-life pavement [5,6]. This issue is exacerbated under frequent extreme weather conditions and increasing traffic volumes, which accelerate asphalt aging through combined environmental and mechanical stresses [7]. The aged asphalt pavement that loses its viscoelasticity is more prone to cracks, peeling, and potholes, which not only seriously affect driving safety but also significantly increase the cost of road maintenance [8,9]. Consequently, developing effective anti-aging technologies for asphalt remains a critical technical requirement for extending the service life of road infrastructure.

Currently, asphalt anti-aging technologies primarily rely on the incorporation of external additives, such as antioxidants [10,11], ultraviolet (UV) absorbers [12,13], and nanomaterials [13,14,15]. For instance, Zhao et al. demonstrated that cerium oxide nanoparticles improve the aging resistance of asphalt mainly through UV absorption [16]. However, the high cost of such nanoparticles remains a significant drawback. Pang et al. found that layered double hydroxides (LDHs) can retard the formation of carbonyl groups during asphalt aging [17], yet the poor compatibility of these inorganic materials with asphalt limits their effectiveness. To simultaneously enhance both thermo-oxidative and UV aging resistance, Chen et al. combined antioxidants with a UV-shielding material, achieving promising radical-scavenging ability and UV-blocking performance [18]. Nevertheless, the complexity of this composite preparation hinders its large-scale production and practical application.

In conclusion, the existing anti-aging additives have slowed down the aging process of asphalt to some extent. However, issues such as high costs, poor compatibility, and the introduction of more complex construction processes still cannot be ignored [19]. An anti-aging solution that does not raise costs or complicate the material system would therefore hold far broader application prospects. On another front, red mud disposal has emerged as a global environmental challenge alongside the rapid expansion of the aluminum industry [20,21]. Its stockpiling not only occupies substantial land but also leads to soil and water contamination due to the alkaline components it contains [22,23]. Consequently, the effective utilization and environmentally sound treatment of red mud have attracted considerable research attention [24,25]. Road engineering has long served as a viable outlet for the consumption of waste materials [26,27]. In this context, a growing number of studies in recent years have explored the use of red mud as a substitute for conventional limestone powder in asphalt pavement filler applications [28].

Studies generally conclude that red mud enhances both the high-temperature performance and adhesive properties of asphalt [29]. Further research indicates that red mud can significantly improve the thermal stability of base asphalt and positively influence the fatigue resistance and moisture susceptibility of asphalt mixtures [30]. In this process, investigations reveal that while red mud improves the high- and low-temperature performance of asphalt binder, its porous structure and surface activity may also influence the aging process of asphalt through physicochemical interactions [31]. Moreover, mineralogical analysis of red mud suggests that its unique components, such as Fe_2_O_3_ and Al_2_O_3_, may possess dual functionality for both UV shielding and radical scavenging [32]. In other words, red mud could also serve as a potential anti-aging agent for asphalt. In summary, red mud forms an effective adsorption interface within asphalt, significantly enhancing the viscosity and high-temperature stability of the binder while also holding promise as an anti-aging material. However, its effectiveness in improving asphalt resistance to thermo-oxidative and ultraviolet aging remains unclear, and the underlying anti-aging mechanisms require further investigation.

Therefore, the primary objective of this study is to systematically evaluate the effect of red mud on the aging resistance of asphalt and to elucidate the underlying mechanisms. First, the microstructural and pore characteristic differences among Bayer-process red mud, sintered red mud, and conventional limestone powder were comparatively analyzed. Subsequently, modified asphalt binders were prepared using each of these three fillers, with commercial antioxidant and UV absorber serving as reference modifiers. The influence of red mud on the aging resistance of asphalt was assessed through conventional physical tests, viscosity measurements, and dynamic rheological analysis. Furthermore, Fourier transform infrared spectroscopy, gel permeation chromatography, and ultraviolet-visible spectrophotometry were employed to reveal the mechanisms by which red mud enhances the anti-aging performance of asphalt. The outcomes of this research are expected to provide a theoretical foundation for the high-value and large-scale utilization of red mud in sustainable road engineering.

## 2. Materials and Methods

### 2.1. Raw Materials

#### 2.1.1. Asphalt

The 70# petroleum asphalt widely employed in China was selected as the virgin asphalt (VA) in this work. Its fundamental properties were tested in accordance with the Chinese specification JTG 3410-2025 [33], and the results are summarized in Table 1.

#### 2.1.2. Fillers

**Figure 1:**
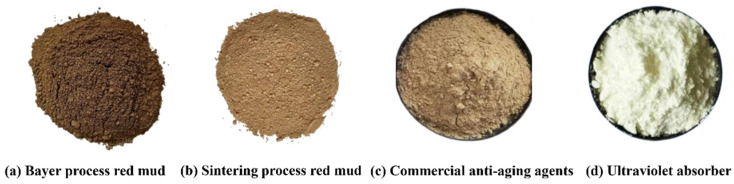
Appearance of red mud, commercial anti-aging agents, and UV absorbers.

Limestone powder (LP) was obtained from the Wenzu Quarry in Jinan City. Both Bayer-process red mud (BM) and sintering-process red mud (SM) were supplied by the Shandong Branch of China Aluminum. The particle size distribution results of the three fillers as measured by a laser particle size analyzer (LS230, Beckman Coulter, Brea, CA, USA) are shown in Table 2. Additionally, a commercially available asphalt anti-aging agent and an ultraviolet absorber (UV-531) supplied by the Nanjing Milan Chemical Co., Ltd. were employed as modifiers to prepare the control asphalt samples. The visual appearance of the red mud, commercial anti-aging agents, and ultraviolet absorbers is presented in Figure 1.

### 2.2. Preparation of Samples

Modified asphalt samples were prepared by incorporating three different fillers and two commercial additives separately. The preparation process was as follows: First, 300 g of the virgin asphalt was heated to 160 °C. Subsequently, the pre-dried fillers and commercial additives were gradually added to the molten asphalt under continuous stirring. The filler content was set at 2%, 4%, and 6% by mass of asphalt for each of the three fillers. Based on preliminary experiments, the dosage of the commercial anti-aging agent and the UV absorber was fixed at 4.0% and 0.4% by mass of asphalt, respectively. A total of 11 types of modified asphalt were thus prepared, each weighing approximately 300 g. For clarity, the abbreviations and material composition information of these asphalt samples are presented in Table 3.

All asphalt samples underwent both thermal-oxidative aging and ultraviolet (UV) aging. Thermal-oxidative aging was simulated using a thin-film oven (SYD-3061, Meiyu Instrument Technology Co., Ltd., Shanghai, China) at 163 °C for 5 h (JTG 3410-2025). Subsequently, to simulate UV-induced aging during service, the samples were subjected to accelerated UV aging by continuous exposure to high-intensity UV light (GHKJ-ZWL, Guanhe Instrument Co., Ltd., Cangzhou, China) at 55 °C for 7 days.

### 2.3. Test Methods

All conventional physical tests were conducted in triplicate, and the reported results represent the mean values. For rheological, FTIR, GPC, and UV-vis analyses, at least two replicates were performed to ensure reproducibility.

#### 2.3.1. Scanning Electron Microscopy

The microscopic morphologies of limestone powder and the two types of red mud were examined using a (SU8000, Hitachi Ltd., Tokyo, Japan) scanning electron microscope operated at an accelerating voltage of 15 kV.

#### 2.3.2. BET-BJH Analysis

The specific surface area and pore characteristics of the three fillers were quantitatively evaluated via nitrogen adsorption–desorption measurements using a dynamic vapor adsorption system (ASAP 2460, Micromeritics Instrument Corporation, Norcross, GA, USA). The specific surface area was calculated from the low-pressure region using the Brunauer–Emmett–Teller (BET) method (Equation (1)). Pore size distribution and pore volume were derived from the adsorption branch in the medium-pressure region using the Barrett–Joyner–Halenda (BJH) model based on capillary condensation theory.

(1) $$SSA^{1/4}=(\frac{n_{m}N_{a}}{M})\alpha$$

where *SSA* is the specific surface area (m^2^/g); *n_m_* is the amount of vapor adsorbed per unit area (mg); *N_A_* is Avogadro’s number (6.022 × 10^23^ mol^−1^); *M* is the molar mass of the vapor molecule (g/mol); *α* is the cross-sectional area of the vapor molecule (m^2^).

#### 2.3.3. Penetration, Softening Point, and Ductility

The influence of red mud on the conventional properties of asphalt was assessed through penetration (at 25 °C), ductility (at 15 °C), and softening point tests. The experiments were conducted in accordance with the Chinese standard JTG 3410-2025.

#### 2.3.4. Rotary Viscosity

The viscosity of asphalt before and after aging was measured with a Brookfield rotational viscometer (DV2THB, Brookfield, MA, USA) at 135 °C, following the standard test method specified in JTG 3410-2025.

#### 2.3.5. Dynamic Shear Rheometer

Frequency sweep tests (JTG 3410-2025) were performed on a dynamic shear rheometer (MCR 102e, Anton Paar, Graz, Austria) to evaluate the viscoelastic properties of the asphalt binders. Tests employed a 25 mm parallel-plate geometry with a 1 mm gap. The frequency ranged from 0.1 to 10 Hz at a fixed shear strain of 1%, and temperatures were varied from 30 to 60 °C in 10 °C increments.

#### 2.3.6. Fourier Transform Infrared Spectroscopy

To investigate the mechanism by which red mud improves the anti-aging performance of asphalt, FTIR spectroscopy (Nicolet iS20, Thermo Fisher, Waltham, MA, USA) was used to analyze changes in functional groups before and after aging. Spectra were collected at a resolution of 4 cm^−1^ over the range of 300–2000 cm^−1^, averaging 32 scans per sample.

#### 2.3.7. Gel Permeation Chromatography

Gel permeation chromatography (Waters LC-1515, Waters, Milford, MA, USA) was employed to analyze the adsorption of asphalt components at different depths on the red-mud surface after stratified extraction. Samples were dissolved in tetrahydrofuran, and Cirrus GPC software (Cirrus v3.3) was used to determine the number-average molecular weight, weight-average molecular weight, and polydispersity index.

#### 2.3.8. Ultraviolet-Visible Absorption Spectrum

The ultraviolet absorption and reflection characteristics of red mud and asphalt were examined by UV-vis spectroscopy (UV-3600, Shimadzu Corporation, Kyoto, Japan). Spectra were recorded in full-wavelength scanning mode from 250 to 500 nm with a data-point resolution of 1 nm.

## 3. Results and Discussion

### 3.1. Properties of Fillers

#### 3.1.1. Microtopography

**Figure 2:**
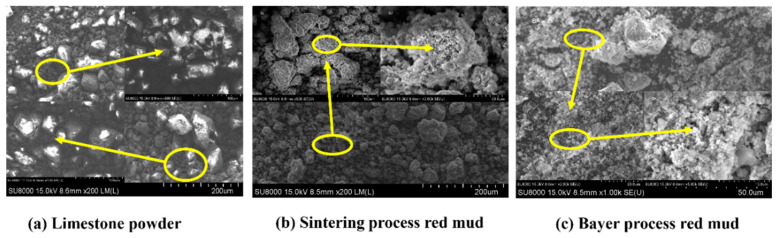
The microscopic morphology of limestone powder and red mud.

The micro-morphologies of LP, BM, and SM were characterized using SEM, as shown in Figure 2. These three materials yield different grain morphologies. Limestone powder particles display a comparatively smooth surface with the sizes presented a homogeneous distribution in the range of 10–100 μm. In contrast, sintered red mud exhibits a layered or flaky structure alongside irregular particles. During the sintering process, certain mineral phases in the sintered red mud undergo high-temperature phase transformation, developing stable layered or plate-like structures through crystal growth [34]. Meanwhile, the hydration of cementitious phases produces fine, porous hydration products, contributing to the overall porosity of this material. Bayer-process red mud consists of spherical aggregates formed by the agglomeration of fine particles. Therefore, its particle size distribution appears to be wider, which is in line with the particle size distribution results shown in Table 2. Bayer-process red mud is a slurry residue formed after the strong alkaline digestion of bauxite. The surface tension of the alkaline medium facilitates the formation of spherical particles, which further aggregate via van der Waals forces during subsequent filtration and sedimentation [35]. Overall, both red mud types display porous microstructures composed predominantly of irregular particles, which establishes a physical basis for their adhesion to asphalt [24].

#### 3.1.2. Porosity Character

**Figure 3:**
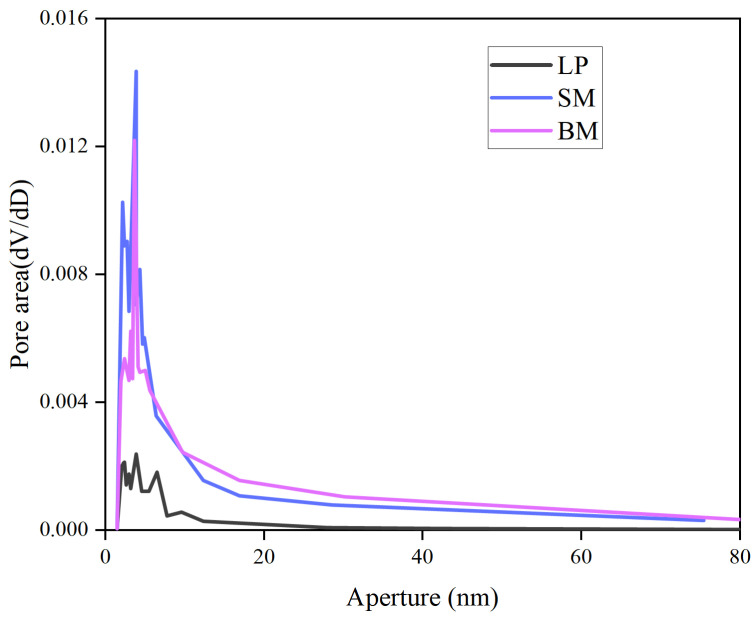
Pore size distribution curves of limestone powder and red mud.

Figure 3 presents the pore size distribution curves of limestone powder and the two red mud types. As illustrated in Figure 3, the pores of limestone powder are predominantly concentrated within 2–10 nm, whereas both sintered and Bayer-process red mud exhibit a broader pore distribution, spanning approximately 2–80 nm. The curve for sintered red mud is notably higher, reflecting a greater number of pores and a wider size range [36]. The corresponding pore volume, average pore diameter, and specific surface area are summarized in Table 4. In terms of pore volume and specific surface area, the values follow the order sintered red mud > Bayer-process red mud > limestone powder, while the average pore diameter remains comparable among the three fillers. Based on these physical characteristics, both types of red mud possess a significantly stronger adsorption capacity for asphalt than limestone powder, which can be attributed to their larger pore volume and specific surface area.

### 3.2. Properties of Modified Asphalt After Thermal-Oxygen Aging

#### 3.2.1. Physical Properties

The effect of red mud on the resistance of asphalt to thermal-oxygen aging was evaluated by comparing the changes in softening point and viscosity before and after aging. The softening point increment (*SPI*) and viscosity aging index (*VAI*) were used as evaluation indices, calculated according to Equations (2) and (3), respectively.

(2) $$SPI_{1}=SPST-SPS$$

(3) $$VAI_{1}=\frac{VSAT-VS}{VS}\times 100\%$$

where *SPST* and *VSAT* represent the softening point and viscosity of asphalt after thermal oxidation; *SPS* and *VS* represent the softening point and viscosity of asphalt before aging.

**Figure 4:**
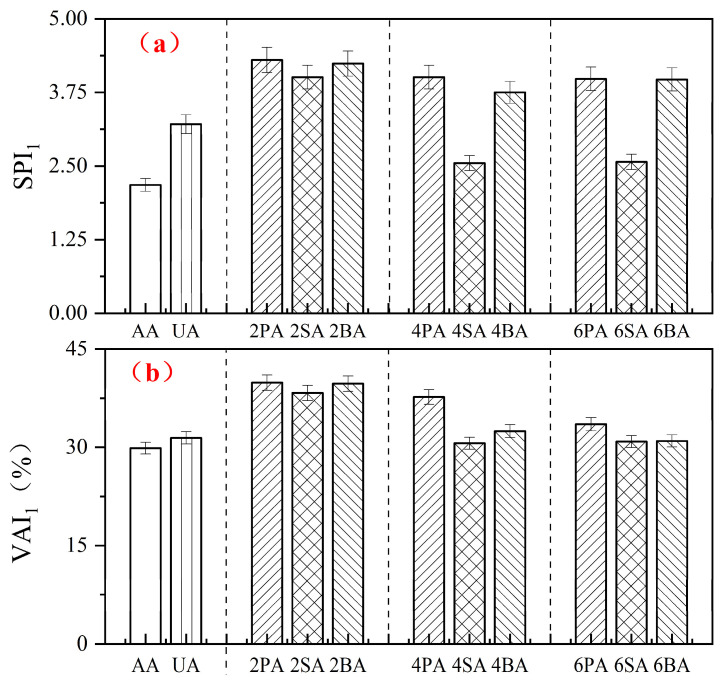
The *SPI*_1_ (**a**) and *VAI*_1_ (**b**) of modified asphalt before and after thermal oxidative aging.

The softening point increment (*SPI*_1_) and viscosity aging index (*VAI*_1_) after thermo-oxidative aging are presented in Figure 4. During thermo-oxidative aging, the combined effect of elevated temperature and oxygen promotes the conversion of light components into heavier components, leading to an increase in both the softening point and viscosity of asphalt. As the content of each filler increases, the values of *SPI*_1_ and *VAI*_1_ gradually decrease, indicating that a higher filler content improves the high-temperature stability of asphalt. This trend is consistent with previous findings by Xiao [37], who reported that increasing red mud content (0–5% by weight of asphalt) resulted in lower *SPI* and *VAI* values after aging.

Notably, at a 4% dosage of SA, the *VAI* and *SPI* reach 27.65 and 3.55, respectively, approaching the performance level achieved with a commercial anti-aging agent. At the same filler dosage, SA exhibits the lowest *SPI*_1_ and *VAI*_1_ among all formulations. The *SPI* of 4SA is 36.3% lower than that of 4PA, and VSI is 18.7% lower. Specifically, paired *t*-tests revealed that the *SPI*_1_ and *VAI*_1_ of 4SA were significantly lower (*p* < 0.05) than those of 4PA and 4BA. This statistical evidence demonstrates that sintered red mud is the most effective filler for preserving the viscoelastic balance of asphalt after thermo-oxidative aging.

The abundant pore structure and high specific surface area of the red mud are likely the main reasons for this effect, as they impede the conversion of light components into heavier fractions in the asphalt. On the other hand, the behavior may be associated with the interfacial interactions between polar oxides in red mud (e.g., Al_2_O_3_’Fe_2_O_3_) and polar functional groups in asphalt, which strengthen cohesion and thermal resistance [24].

#### 3.2.2. Rheological Properties

In this section, the phase angle aging index (*PAAI*) and the complex modulus aging index (*CMAI*) were introduced to evaluate the effect of red mud on the aging resistance of asphalt. The calculation formulas are presented as Equations (4) and (5), respectively.

(4) $$PAAI=\frac{\delta }{\delta_{0}}$$

(5) $$CMAI=\frac{G}{G_{0}}$$

where *δ* and *δ*_0_ represent the phase angle of asphalt before and after thermal oxidation; *G* and *G*_0_ represent the complex modulus of asphalt before and after aging.

**Figure 5:**
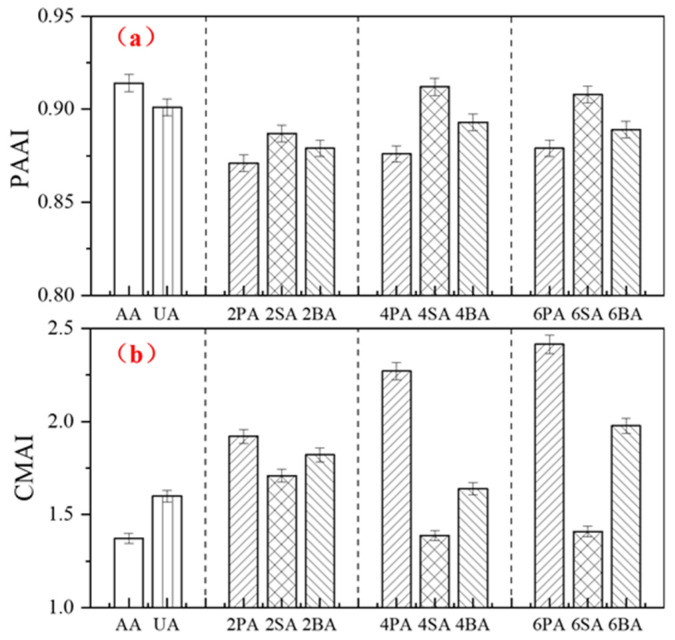
The *PAAI* (**a**) and *CMAI* (**b**) of modified asphalt before and after thermal oxidative aging.

Figure 5 presents the phase angle aging index (*PAAI*) and complex modulus aging index (*CMAI*) of asphalt after thermo-oxidative aging. Aging resulted in a decreased phase angle and increased complex modulus, indicating an enhanced elastic response and improved deformation resistance of the material. Specifically, the *PAAI* of 4SA was 4.1% higher than that of 4PA and 2.1% higher than that of 4BA, while its *CMAI* was 38.9% lower than that of 4PA and 15.3% lower than that of 4BA. Paired *t*-tests confirmed that SA exhibited statistically significant differences from both BA and PA for both indicators (*p* < 0.05).

At the same filler content, both types of red mud improved the aging resistance of asphalt, with SA demonstrating superior performance to BA. Notably, the aging indices of asphalt containing 4% SA approached those achieved with a commercial anti-aging agent. However, at a 6% dosage, no significant further improvement was observed, likely due to particle agglomeration and poorer dispersion of red mud in the asphalt matrix at higher concentrations [38]. These findings confirm that asphalt containing 4% sintered red mud exhibits the most pronounced resistance to thermo-oxidative aging, consistent with the results discussed in previous sections.

#### 3.2.3. Functional Group

**Figure 6:**
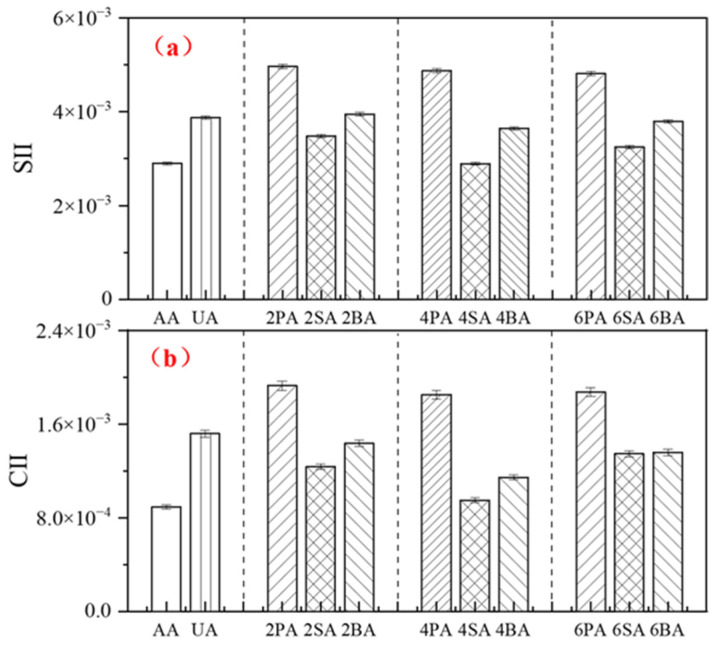
The *CII* (**a**) and *SII* (**b**) of modified asphalt before and after thermal oxidative aging.

The effect of red mud on the aging resistance of asphalt was evaluated using the carbonyl index increment (*CII*) and sulfoxide index increment (*SII*). The calculation formulas are given in Equations (6) and (7), respectively. The results are shown in Figure 6.

(6) $$CII=CI-CI_{0}=\frac{A_{1700^{{\mathrm{cm}}^{-1}}}}{A_{2000-600^{{\mathrm{cm}}^{-1}}}}$$

(7) $$SII=SI-SI_{0}=\frac{A_{1030^{{\mathrm{cm}}^{-1}}}}{A_{2000-600^{{\mathrm{cm}}^{-1}}}}$$

where *CI*_0_ and *SI*_0_ represent the carbonyl index and sulfoxide index of the asphalt before aging; *CI* and *SI* represent the carbonyl index and sulfoxide index of the asphalt after aging; *A* is the band area of the relevant absorption band in the FTIR spectrum.

At the same filler dosage, the carbonyl index increment (*CII*) and sulfoxide index increment (*SII*) of PA were significantly higher than those of SA and BA at the *p* < 0.05 level, indicating that the incorporation of red mud effectively suppresses the formation of oxygen-containing functional groups during asphalt aging, thereby enhancing its aging resistance. This finding is consistent with previous research, which reported that the *CII* of neat asphalt increased by 0.00262 after thermal oxidative aging, whereas the increase was only 0.0016 for asphalt containing 5% sintered red mud [36]. Among all samples, the commercial anti-aging agent (AA) exhibited the smallest increments in *CII* and *SII*. Notably, the increments for asphalt containing 4% SA were lower than those for 4% BA and approached the level of AA. These results further confirm that sintered red mud provides the most significant enhancement in the resistance of asphalt to thermo-oxidative aging.

### 3.3. Properties of Modified Asphalt After UV Aging

The fundamental distinction between UV aging and thermal-oxidative aging lies in the mechanisms that drive chemical bond cleavage. Thermal-oxidative aging primarily results from the combined action of elevated temperature and oxygen, which promotes the thermal decomposition of chemical bonds in the asphalt. In contrast, UV aging is initiated when chromophores in the material absorb ultraviolet radiation, transition to an excited state, and subsequently induce bond dissociation. Moreover, oxidation products generated under UV irradiation are often reactive and tend to undergo further secondary reactions, thereby accelerating the overall aging process. In this study, sequential aging—first by thermo-oxidative and then by UV exposure—was adopted to simulate the combined aging effects encountered under actual service conditions. The corresponding changes in asphalt performance after aging were measured and systematically analyzed.

#### 3.3.1. Physical Properties

**Figure 7:**
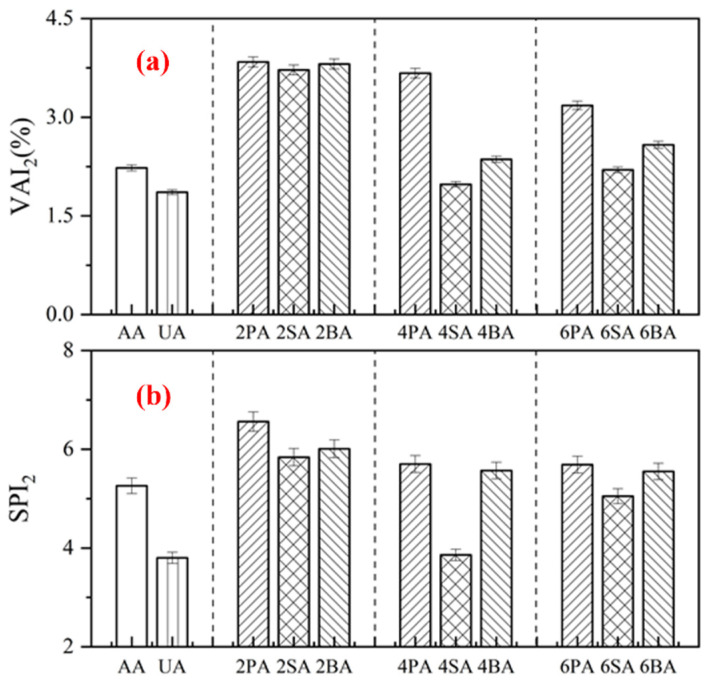
The *SPI_2_* (**a**) and *VAI_2_* (**b**) of modified asphalt before and after UV aging.

As shown in Figure 7, after UV aging, asphalt modified with LP exhibited the highest softening point increment (*SPI*_2_) and viscosity aging index (*VAI*_2_), indicating its poorest resistance to UV aging. At a 2% filler dosage, the *SPI*_2_ and *VAI*_2_ values of PA, SA, and BA were comparable, suggesting that a low filler content provides limited improvement in UV aging resistance. When the sintered red mud content was increased to 4%, its *VAI*_2_ and *SPI*_2_ reached 1.98% and 4.86%, respectively—values that approached those achieved with the commercial anti-UV aging agent.

Specifically, the *VAI*_2_ of 4SA was 85.4% lower than that of PA and 16.1% lower than that of BA, while its *SPI*_2_ was 32.3% lower than that of PA and 30.9% lower than that of BA. At the same dosage, SA exhibits the lowest *SPI*_2_ and *VAI*_2_ values. Paired *t*-test revealed that both the *SPI* and *VAI* of 4SA were significantly lower than those of 4PA and 4BA (*p* < 0.05), providing statistical evidence that sintered red mud offers the most effective resistance to ultraviolet aging in asphalt. This superior performance can be attributed to the abundant pore structure of SA, which effectively absorbs UV radiation [39].

#### 3.3.2. Rheological Properties

**Figure 8:**
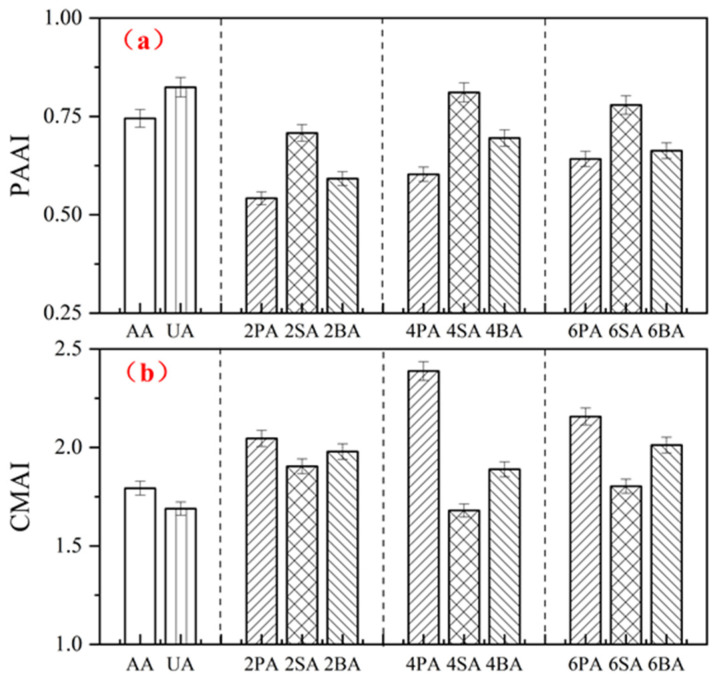
The *PAAI* (**a**) and *CMAI* (**b**) of modified asphalt before and after UV aging.

Figure 8 presents the phase angle aging index (*PAAI*) and complex modulus aging index (*CMAI*) of asphalt after UV aging. As shown in Figure 8a, UV exposure results in a marked decrease in *PAAI*, indicating a reduction in the material’s elastic response. At the same filler dosage, SA exhibits the highest *PAAI*, demonstrating that sintered red mud is the most effective in preserving the viscoelastic balance of asphalt after UV aging. Notably, the *PAAI* of SA at a 4% dosage reaches 0.812, approaching the level achieved with the commercial ultraviolet absorber. Specifically, the *PAAI* of 4SA is 44.4% higher than that of 4PA and 16.7% higher than that of 4BA. Paired *t*-tests further confirmed that the *PAAI* of SA-4% was significantly different from both PA-4% and BA-4% (*p* = 0.0029, *p* = 0.0029), providing statistical evidence for its superior UV aging resistance.

Figure 8b shows that the complex modulus increases substantially after UV aging. PA exhibited the highest *CMAI*, further confirming its inferior resistance to UV aging. At the same dosage, the *CMAI* of SA was consistently lower than that of BA, confirming that sintered red mud provides superior UV aging resistance to Bayer-process red mud. Specifically, the *CMAI* of 4SA was 29.6% lower than that of 4PA and 11.1% lower than that of 4BA. The *CMAI* of the ultraviolet absorber (UA) was slightly higher than that of 4% SA, a trend consistent with the *PAAI* results. Together, both the *PAAI* and *CMAI* results suggest that 4% sintered red mud offers the most effective mitigation of UV-induced aging. In contrast, the commercial anti-aging agent (AA) showed limited improvement, likely because its exfoliated phase structure does not provide an adequate barrier against ultraviolet radiation, thus failing to significantly enhance the UV aging resistance of the asphalt.

#### 3.3.3. Functional Group

**Figure 9:**
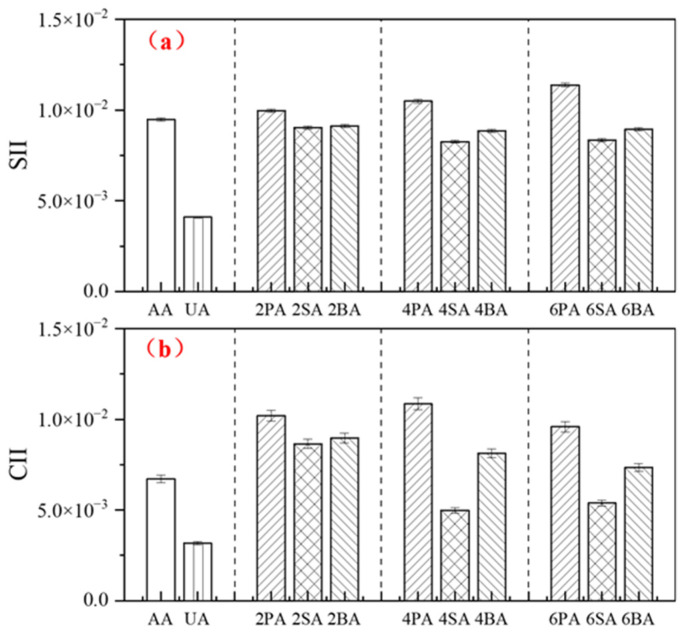
The *CII* (**a**) and *SII* (**b**) of modified asphalt before and after UV aging.

The changes in the *SII* and *CII* of asphalt after UV aging are shown in Figure 9. Among all samples, the ultraviolet absorber (UA) exhibits the lowest *SII* and *CII* values, indicating the best UV aging resistance [40]. In contrast, limestone powder (PA) yielded the highest values, indicating the poorest aging resistance. At the same dosage, the sintered red mud (SA) presented lower *SII* and *CII* values than the Bayer-process red mud (BA), confirming its superior ability to mitigate UV-induced oxidation. Notably, the 4% SA dosage not only delivered the optimal UV aging resistance among the fillers but also showed good thermo-oxidative aging resistance, as shown in earlier sections.

Statistical analysis using paired *t*-tests revealed that all key aging indicators—including *SPI*, *VAI*, *CMAI*, *PAAI*, *CII*, and *SII*—showed statistically significant differences for 4SA-4% compared to the control groups (4PA and 4BA), with all *p*-values below 0.05. These findings statistically validate that sintered red mud at a 4% content holds promising potential as an effective anti-aging additive for asphalt.

### 3.4. Analysis of the Anti-Aging Mechanism

#### 3.4.1. FTIR Analysis

**Figure 10:**
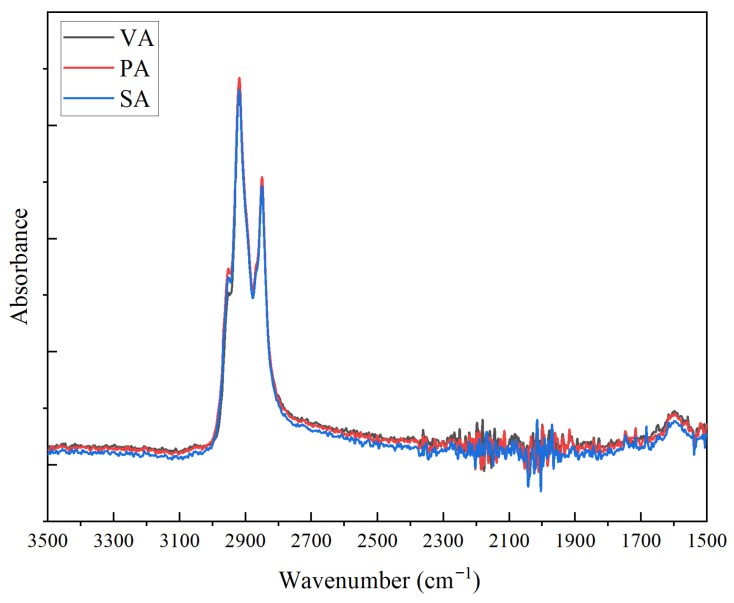
Infrared spectrum of modified asphalt.

The functional group changes in the virgin asphalt with the addition of limestone powder or sintered red mud were characterized by Fourier Transform Infrared (FTIR) spectroscopy. Variations in the absorption bands reflect changes in the relative content of functional groups in the asphalt. Figure 10 indicates that no new absorption bands or band shifts were observed after the incorporation of either filler, suggesting that the interaction between asphalt and fillers is primarily physical. Nevertheless, the intensity of the characteristic absorption bands changed noticeably; the corresponding numerical data are summarized in Table 5.

Compared with the virgin asphalt, the addition of limestone powder or sintered red mud resulted in a notable decrease in the intensity of absorption bands at 3440 cm^−1^, 2930 cm^−1^, 2850 cm^−1^, and 1600 cm^−1^. This indicates a reduction in the relative content of hydrogen bonds, alkyl chains, cyclic structures, and aromatic functional groups in the modified asphalt [41]. Among these, the band at 3440 cm^−1^, associated with intermolecular hydrogen bonds, showed the most pronounced attenuation in the sintered red mud modified asphalt, with a reduction of 19.8% decrease relative to the base asphalt, which is attributed primarily to molecular-chain scission induced by high-shear mixing.

Furthermore, SA exhibited the lowest response value at 2930 cm^−1^, and its absorption at 2850 cm^−1^ was also lower than that of the virgin asphalt across all systems, reflecting its reduced alkane content. Additionally, the incorporation of sintered red mud led to a pronounced decrease in the intensity of the band at 1600 cm^−1^, characteristic of aromatic compounds. Combined with the SEM morphology analysis, these results suggest that the layered surface of sintered red mud selectively adsorbs portions of saturates and aromatics from the asphalt.

#### 3.4.2. GPC Analysis

**Figure 11:**
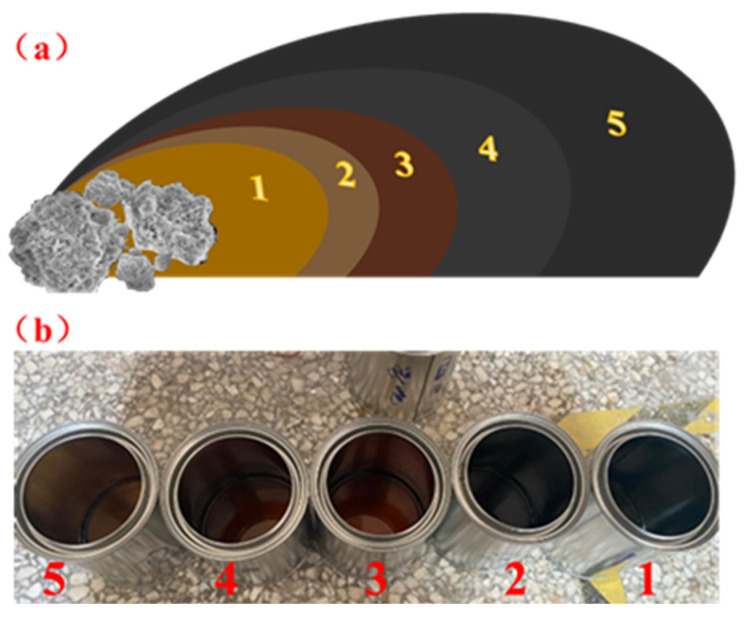
Schematic diagram of extracting structural asphalt by toluene (**a**) and the five fractions obtained (**b**).

To investigate the selective adsorption of asphalt components by red mud, a surface adsorption model experiment was designed. Asphalt was first dissolved in toluene to prepare a homogeneous solution. The solution was then filtered through a vacuum filtration setup to remove the toluene solvent and unbound asphalt, retaining the bound asphalt adsorbed on the red mud particles, thus obtaining red mud–asphalt composite particles. The particles were subsequently subjected to five successive elutions, yielding five fractions of eluted asphalt-toluene solution, as shown in Figure 11. The total mass of the filter paper, red mud, and adsorbed asphalt was measured separately to determine changes in asphalt film thickness and adsorbed mass. After solvent removal by distillation, five asphalt samples corresponding to the different elution layers were obtained.

**Figure 12:**
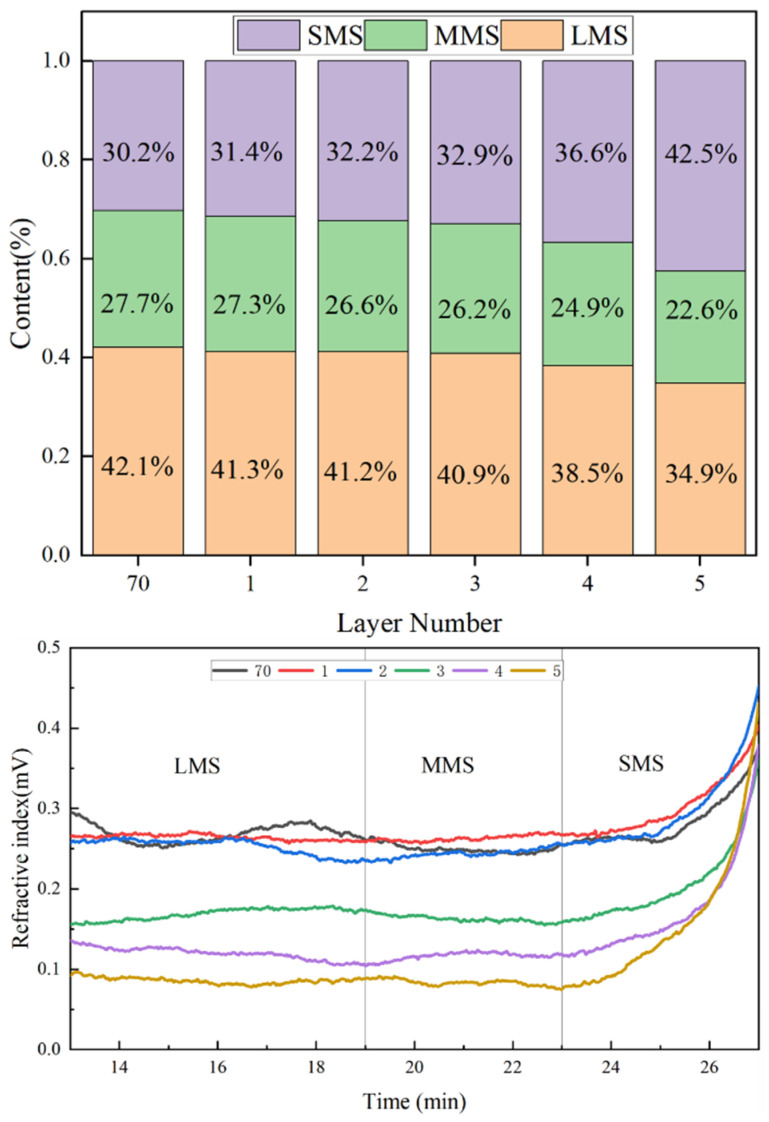
The results of the GPC experiment for toluene elution of asphalt.

The five eluted asphalt samples were analyzed by gel permeation chromatography (GPC). Figure 12 presents the proportions of large (LMS), medium (MMS), and small (SMS) molecules in each eluted sample relative to the base asphalt. The molecular weight distributions of samples 1 and 2 were essentially consistent with that of the base asphalt, which is approximately around 30%. As the sample number increased (i.e., for layers closer to the red mud surface), the proportion of small molecules rose significantly. The SMS content of Sample 5 has reached as high as 42.5%. The research results indicate that there is a relatively high concentration of light components near the surface of the red mud, and the red mud has the ability to selectively adsorb the light components in the asphalt [42].

The elution profiles in Figure 12 show that during the 14–27 min retention period, the chromatographic bands systematically shifted to longer retention times with successive elution, indicating that smaller molecules exhibit longer retention. Furthermore, samples closer to the red mud particle surface (higher layer numbers) contained lower amounts of large and medium molecules and higher amounts of small molecules, confirming that the asphalt retained within the red mud is predominantly composed of light components. These results demonstrate that red mud possesses a distinct preferential adsorption capacity for the light fractions of asphalt.

#### 3.4.3. Ultraviolet-Visible Absorption Spectroscopy Analysis

**Figure 13:**
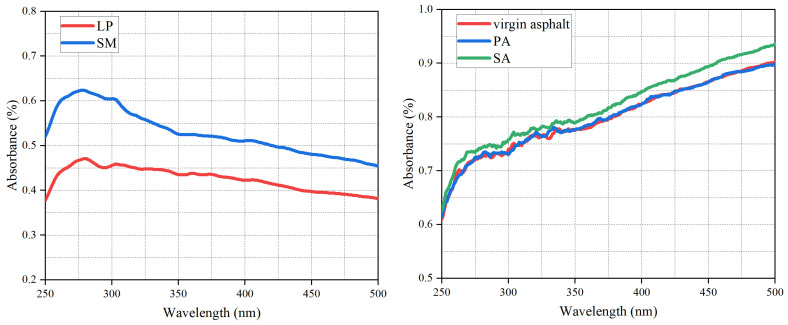
Absorption of UV by LP, SM, and their modified asphalt.

To investigate the mechanism by which red mud enhances the UV aging resistance of asphalt, ultraviolet-visible (UV-Vis) absorption spectroscopy was employed to analyze the ultraviolet absorption intensity and transmittance of the fillers and their corresponding asphalt mastics. As shown in Figure 13, the absorbance of sintered red mud for UV more effectively across the tested wavelengths than limestone powder. For example, the absorbance of sintered red mud is 0.60% for 300 nm UV, which is significantly higher than that of limestone powder of 0.45% for 300 nm UV, indicating that its uniform dispersion in asphalt can effectively absorb UV radiation [36]. Consistent with this, the asphalt mastic containing sintered red mud exhibited the highest UV absorption (0.76% for 300 nm UV), whereas the absorption of the limestone powder-modified asphalt was limited and similar to that of the base asphalt (0.73% for 300 nm UV).

**Figure 14:**
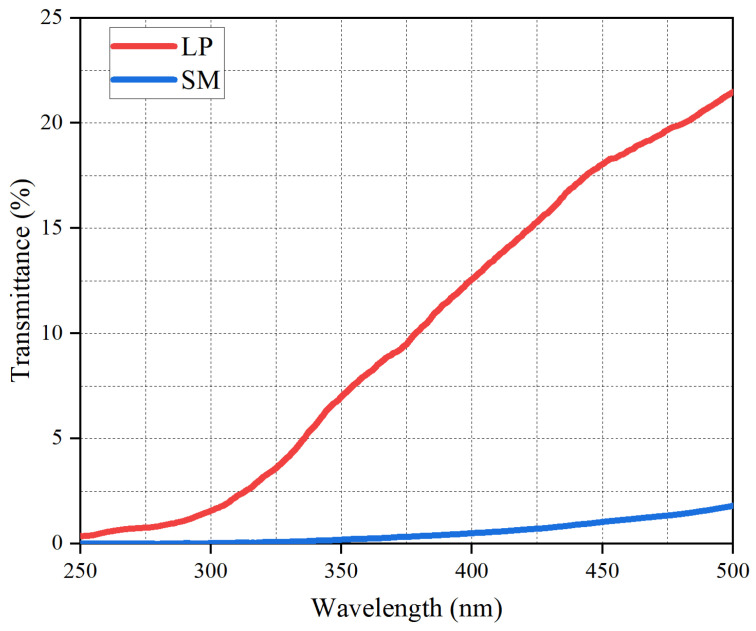
The transmittance of LP and SM.

Figure 14 presents the UV transmittance of limestone powder and sintered red mud. Sintered red mud transmitted UV radiation weaker across the tested wavelengths than limestone powder. For example, the transmittance of sintered red mud to UV with a wavelength of 400 nm is 1%, while that of LP is as high as 12.5%. The results indicate that the transmittance of sintered red mud is markedly lower. Integrated with the absorption data in Figure 13, it is evident that sintered red mud provides superior UV shielding within the asphalt compared to limestone powder.

**Figure 15:**
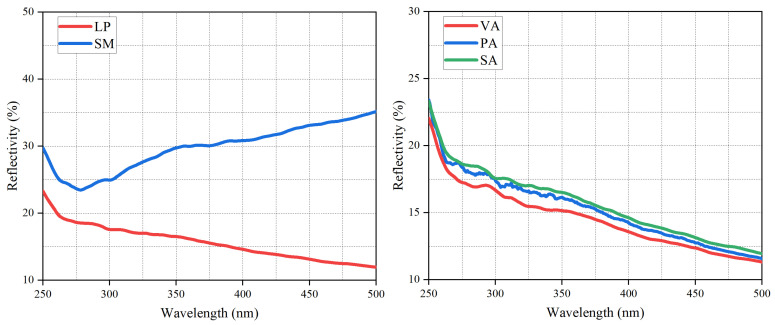
The reflectance of LP, SM, and their modified asphalt.

Furthermore, the UV reflectance results are shown in Figure 15. Sintered red mud reflected UV radiation more effectively across the tested wavelengths than limestone powder, which can be attributed to its larger specific surface area and abundant pore structure that promote light scattering. Additionally, the UV reflection results of red mud in this study are largely consistent with those mentioned in the literature: there is a turning point at 275 nm, and then the reflection value gradually increases as the wavelength increases [36]. For example, the reflectance of sintered red mud to UV with a wavelength of 400 nm is 31.0%, while that of LP is 14.0%. Correspondingly, the asphalt mastic with sintered red mud exhibited the highest UV reflectance. In summary, based on the comprehensive analysis of absorbance, transmittance, and reflectance, the incorporation of sintered red mud effectively delays the aging process of asphalt by reducing UV penetration and enhancing light reflection, thereby significantly improving its resistance to UV aging during service.

## 4. Conclusions

Based on the experimental results and subsequent analysis, the main conclusions of this study are summarized as follows:

(1) Microstructural and pore-structure analyses reveal that both types of red mud possess rougher surface morphologies, more developed pore networks, and significantly higher specific surface areas and pore volumes compared with limestone powder. The particle size distribution of red mud is wider than that of limestone due to its agglomeration.
(2) Both types of red mud considerably enhance the resistance of asphalt to thermo-oxidative aging relative to limestone powder. At a 4% dosage, the improvement offered by sintered red mud is comparable to that achieved with a commercial antioxidant. Similarly, red mud substantially improves the UV aging resistance of asphalt. At 4% content, sintered red mud performs similarly to a commercial UV absorber.
(3) FTIR and GPC results indicate that the layered surface of sintered red mud selectively adsorbs low-molecular-weight asphalt components, such as saturates and aromatics, which contribute to its ability to mitigate thermo-oxidative aging. Moreover, UV-vis spectroscopy confirms that sintered red mud significantly reduces ultraviolet transmittance and increases light reflection, thereby enhancing the UV aging resistance of asphalt.
(4) Statistical analysis using paired *t*-tests revealed that all key aging indicators including *SPI*, *VAI*, *CMAI*, *PAAI*, *CII*, and *SII* showed statistically significant differences for 4SA compared to the control groups, with all *p*-values below 0.05. These findings statistically validate that sintered red mud at a 4% content holds promising potential as an effective anti-aging additive for asphalt.

## Figures and Tables

**Table 1:** The fundamental properties of virgin asphalt.

Items	Requirements	Results	Units	Methods
Penetration	60–80	68.5	0.1 mm	T0604
Ductility	≥100	>100	cm	T0605
Softening point	≥46	47.6	°C	T0606
Viscosity (135 °C)	-	0.450	Pa·s	T0625

**Table 2:** The densities and particle size distribution of LP, BM, and SM.

Items	Apparent Density (g/cm^3^)	Particle Size Distribution (μm)		
		Dv (10)	Dv (50)	Dv (90)
LP	2.778	10.69	37.48	113.4
BM	2.852	2.79	93.95	203.8
SM	2.553	13.91	74.67	160.1

**Table 3:** The abbreviations and definitions for asphalt samples used in this study.

Material Composition	Abbreviation
Limestone powder: asphalt = 2:100/4:100/6:100	2PA/4PA/6PA
Sintering process red mud: asphalt = 2:100/4:100/6:100	2SA/4SA/6SA
Bayer process red mud: asphalt = 2:100/4:100/6:100	2BA/4BA/6BA
Commercial anti-aging agents: asphalt = 4:100	AA
Ultraviolet absorber: asphalt = 0.4:100	UA

**Table 4:** Pore parameters of limestone powder and red mud.

Items	Pore Volume (cc/g)	Mean Pore Size (nm)	Specific Surface Area (m^2^/g)
LP	0.019	3.07	9.97
SM	0.125	3.06	30.9
BM	0.098	3.01	30.5

**Table 5:** The infrared absorption band intensity of modified asphalt.

Items	3440 cm^−1^	2930 cm^−1^	2850 cm^−1^	1600 cm^−1^	3440 cm^−1^
VA	0.0024	0.180	0.127	0.0067	0.0024
PA	0.0022	0.177	0.126	0.0038	0.0022
SA	0.0020	0.176	0.126	0.0027	0.0020

## Data Availability

The original contributions presented in this study are included in the article. Further inquiries can be directed to the corresponding author.
